# Supplementary material for: Identification of photoperiod-induced specific miRNAs in the adrenal glands of Sunite sheep (Ovis aries)
Source: Front Vet Sci. 2022 Jul 22;9:888207. doi: 10.3389/fvets.2022.888207 (PMC9354845; doi:10.3389/fvets.2022.888207)
Supplement: Supplementary file 1 [file Data_Sheet_1.ZIP › Supplementary file/miRDeep2 file.zip!/miRDeep2 file/Novel_Structure/SP42A3/Novel_273.pdf]

Provisional ID	:	NC_019470.2_19813
Score total	:	1.8
Score for star read(s)	:	-1.3
Score for read counts	:	0
Score for mfe	:	1.5
Score for randfold	:	1.6
Score for cons. seed	:	
Total read count	:	85
Mature read count	:	85
Loop read count	:	0
Star read count	:	0

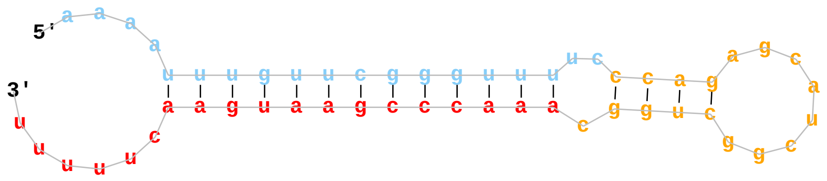

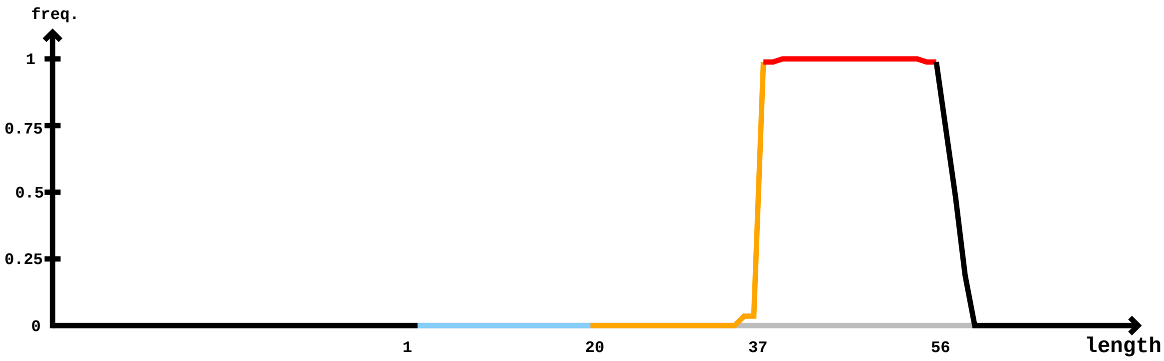

Star Mature

```
5'- guguugaucuaacacacuauaucucagguuggucaaaaaauuuguucggguuuucccagagcaucggcuggcaaacccgaaugaacuuuuuggucaacucacuacucuggc   -3'    exp
    ((((((...))))))...........(((((((((((((.(((((((((((((..((((........)))).))))))))))))).))))))))))))).............   reads     mm      sample
    ......................................................................gcaaacccgaaugaacuuuuu....................   1         0       seq
    ......................................................................gcaaacccgaaugaacuuuuug...................   2         0       seq
    ........................................................................aaacccgaaugaacuuu......................   1         0       seq
    ........................................................................aaacccgaaugaacuuuuu....................   32        0       seq
    ........................................................................aaacccgaaugaacuuuuuC...................   1         1       seq
    ........................................................................aaacccgaaugaacuuuuuA...................   3         1       seq
    ........................................................................aaaccAgaaugaacuuuuug...................   1         1       seq
    ........................................................................aaacccgaaugaacuuuuuU...................   3         1       seq
    ........................................................................aaacccgaaugaacuuuuugg..................   21        0       seq
    ........................................................................aaacccgaaugaacuuuuuUg..................   4         1       seq
    ........................................................................aaacccgaaugaacuuuuuggu.................   15        0       seq
    ..........................................................................acccgaaugaacuuuuuggu.................   1         0       seq
```
